# A national survey on the use of ultrasound as an educational tool to complement anatomy teaching at UK medical schools

**DOI:** 10.1002/ca.24229

**Published:** 2024-09-26

**Authors:** Ansh Tandon, Joseph Moneim, Lauren Hector, Peter Fletcher, Ishpal Moonga, Sarah Fawcett, Helen Taylor, Cecilia Brassett

**Affiliations:** ^1^ Human Anatomy Centre, Department of Physiology, Development and Neuroscience University of Cambridge Cambridge UK

**Keywords:** anatomy, education, radiology, teaching, ultrasound

## Abstract

Our aim was to investigate the current use of ultrasound (US) in anatomy teaching across the UK, and to discover potential obstacles in its implementation in the undergraduate medical curriculum. An electronic survey consisting of 31 questions was distributed to anatomy course organizers at all 42 UK medical schools from June to October 2022. Both quantitative and qualitative data were obtained. Analysis of quantitative data was performed using frequency tables while responses to open‐ended questions were analyzed individually by authors, and themes were extracted for presentation. There was a 100% response rate, with 23 (55%) medical schools using US in anatomy teaching. Of these, 17 (74%) schools taught normal US anatomy only, while 6 (26%) included pathology. Only 3 (13%) schools delivered weekly sessions, while 3 (13%) schools delivered monthly sessions and 17 (74%) held less frequent sessions. Of the 17 schools with hands‐on sessions, these were delivered by radiologists in 6 schools, while sessions in other schools were facilitated by sonographers, other clinicians, students, and anatomy department staff. Students were able to operate US machines themselves in all 17 schools delivering hands‐on teaching. Limitations in resources and trained staff as well as time constraints were cited as barriers for the introduction of US in anatomy teaching. Our results indicate that only just over half of all UK medical schools use US in anatomy teaching. As US is increasingly used in screening and diagnosis by various clinicians, learning how to use US early in the medical course would be beneficial. Identifying the barriers to introducing US in the anatomical curriculum is the first step towards the successful development of an US teaching programme.

**Abbreviations**
GP: general practitioner
POCUS: point of care ultrasound
US: ultrasound

## 1 INTRODUCTION

A sound knowledge and appreciation of anatomy is required in all medical specialties. It is therefore crucial that medical schools deliver anatomical teaching that provides a robust foundation for its application in future clinical practice. Ultrasound (US) is commonly used in screening programmes, point of care diagnostics, in acute settings by non‐radiologists and to facilitate procedures such as central venous cannulation (Whitson & Mayo, 2016; Saugel et al., 2017; Ferraioli & Soares Monteiro, 2019; ‘Abdominal aortic aneurysm screening: programme overview’, GOV.UK, 2021). In many medical schools in the United States and Continental Europe, US has been integrated into the undergraduate curriculum in recent years (Nicholas et al., 2021; Teichgräber et al., 1996). In light of this, the 2022 Undergraduate Radiology Curriculum from the Royal College of Radiologists indicates that students should be aware of US‐based imaging features of common pathology (The Royal College of Radiologists, 2022).

Recent literature has highlighted debates surrounding the efficacy and optimal methods for incorporating US into medical education curricula (Celebi et al., 2019; Patel et al., 2017; Smith & Barfoot, 2021; Tarique et al., 2018). Areas of discussion have included the pedagogical effectiveness of US in enhancing anatomical understanding, curricular design, delivery, and striking an appropriate balance between theoretical knowledge and practical skills acquisition. When considering hands‐on US teaching, a discussion surrounding governance exists as to whether those who act as US subjects should be scanned in case there are any incidental findings, and this highlights practical issues that must be managed when incorporating US into the curriculum. These debates reflect broader shifts in medical education towards interdisciplinary collaboration and the evolving role of technology in teaching. Fundamentally, the aim of integrating US into teaching is to enhance students' understanding of anatomy and its clinical relevance. Student surveys are positive, with multiple studies showing that students feel US increases their understanding of gross anatomy (Gritton et al., 2019), anatomical concepts, and clinical correlates (Edwards et al., 2023; Lufler et al., 2022). However, the advantages of integrating US into an anatomy course compared to dissection or prosection have not been extensively investigated. Limited evidence suggests that US‐based anatomical teaching may provide an alternative to prosections in specific regions, such as cardiac anatomy (Griksaitis et al., 2012). Some have gone further in integrating US‐based anatomy alongside the early development of clinical skills, including point of care ultrasound (POCUS) (Olivares‐Perez et al., 2022) and US‐guided procedures (Brown et al., 2012). A recent literature review of studies from eight UK medical schools demonstrated that different approaches were used to deliver US teaching (McCormick et al., 2023). Most commonly, this was clinician‐led, hands‐on teaching of predominantly normal anatomy in the early years (1–3) of medical education. Less than half the studies focused on teaching POCUS or US‐guided procedures. McCormick et al. (2023) concluded that while it was possible to adequately train students to perform some POCUS, it was unclear whether this experience would lead to competency in these skills. Currently, only one UK medical school has integrated an US curriculum across their 5‐year programme, which includes all body systems (Wakefield et al., 2018). With increasing use of US in the anatomy curriculum, further analysis will hopefully aid in evaluating how optimal integration can be achieved. Therefore, this survey aimed to investigate the current use of US in UK anatomy teaching and to explore any potential barriers to its implementation.

## 2 MATERIALS AND METHODS

### 2.1 Ethics statement

This study received ethics approval by the Cambridge Human Biology Research Ethics Committee. The ethical approval number is HBREC.2022.10.

### 2.2 Survey format

The survey consisted of 31 questions divided into four themes: (1) Current US usage, (2) Format of US‐based teaching, (3) US teaching faculty, (4) Barriers and challenges. The survey contained multiple choice, Likert scale (with a score of 1 indicating strongly negative and 5 strongly positive), and open‐ended questions. The survey was created specifically for this study in collaboration with clinical anatomists, anatomy demonstrators and radiologists at the University of Cambridge. Due to the small respondent population size, the survey was not piloted prior to distribution.

### 2.3 Survey distribution

The survey was electronically distributed between June and October 2022 to the anatomy course organizers of all 42 UK medical schools as listed by the Medical Schools Council (Medical Schools Council, 2022). In cases where the course organizer did not respond or was inaccessible, the survey was resent to other faculty members. The survey could be accessed via an online platform (Google Forms) or by completing a Word document.

### 2.4 Survey analysis

All responses were collated into an Excel spreadsheet. In view of the 100% response rate from all UK medical schools, descriptive analysis of quantitative data was performed using frequency tables. Responses to open‐ended questions were analyzed individually by authors, with themes extracted and semi‐quantified for presentation.

## 3 RESULTS

### 3.1 Current US usage

Responses were received from all 42 (100%) UK medical schools (Table 1). There are 25 (60%) schools that teach anatomy solely in years 1–2 with an average number of 254 (±105**)** students per year. Anatomy is taught using a variety of resources and methods. There are 36 (86%) medical schools that use didactic lectures, 37 (88%) employ synthetic anatomical models, and 37 (88%) use surface anatomy. There are 23 (55%) medical schools that use US in anatomy teaching. There are 16 (38%) medical schools that examine US anatomy knowledge. Of these 16 schools, 14 use US images in their examinations, comprising on average <5% of all questions.

**TABLE 1:** US teaching in UK medical schools.

Medical school responses	*n* (%)
Years anatomy taught	*n* = 42
Year 1	2 (5)
Year 1–2	23 (55)
Year 1–2+	17 (40)
Average number of students per year	254 ± 105 (*n* = 41)
Anatomical teaching methods	*n* = 42
Cadaveric dissection	22 (52)
Anatomical prosections	34 (81)
Synthetic anatomical models	37 (88)
Surface anatomy	37 (88)
Virtual dissector programmes	14 (33)
Problem‐based learning (PBL)	18 (43)
Didactic lectures	36 (86)
US used in anatomy teaching	23 (55)
Examination of US anatomy	*n* = 16
US images used in exams	14 (88)
US question content	<5%

### 3.2 Format of US‐based teaching

Of the 23 schools that use US, the frequency of US sessions varies: weekly sessions in 3 (13%) schools, monthly in 3 (13%) schools and less frequently in 17 (74%) schools (Table 2). There are 6 (26%) schools that use pre‐recorded US resources only, 4 (17%) run hands‐on practical sessions only, with 13 (57%) using a combination of the two. There are 17 (74%) schools that teach normal US anatomy only, while 6 (26%) schools also include examples of pathology. Of the 17 schools employing hands‐on teaching, tailored instruction handouts are used by 13 (76%) schools.

**TABLE 2:** Format of US based teaching.

	*n* (%)
Frequency of US anatomy teaching	*n* = 23
Weekly	3 (13)
Monthly	3 (13)
Less than monthly	17 (74)
US methods	*n* = 23
Pre‐recorded US resources only	6 (26)
Hands‐on practical sessions only	4 (17)
Pre‐recorded US resources and hands‐on practical sessions	13 (57)
US teaching used to demonstrate	*n* = 23
Normal anatomy only (includes normal variants)	17 (74)
Examples of pathology	6 (26)
Resources used to support hands‐on US teaching	*n =* 17
Tailored instruction handouts	13 (76)
Computer software during sessions	2 (12)
Access to internet resources during sessions	7 (41)
Access to textbooks during sessions	8 (47)

### 3.3 US teaching faculty

Clinical radiologists are involved in the course design of the US‐based anatomy teaching in 15 (65%) of the 23 schools using US. Of the 17 schools employing hands‐on US anatomy teaching, teaching is delivered solely by non‐radiologists in 11 (65%) schools, while 6 (35%) schools use a combination of clinical radiologists and non‐radiologists (Table 3). Non‐radiologists include other medical students, anatomy demonstrators and sonographers. Regarding the US subjects used in hands‐on US teaching, students are used in 12 (71%) schools, faculty members in 5 (29%), professional actors in 5 (29%) and other volunteers in 2 (12%). There are 7 (41%) medical schools that use pre‐screening of their US subjects for pathology prior to teaching sessions.

**TABLE 3:** US teaching faculty.

	*n* (%)
Practical US teaching staff	*n* = 17
Non‐radiologists	11 (65)
Clinical radiologists and non‐radiologists	6 (35)
US subjects	*n* = 17
Students	12 (71)
Faculty	5 (29)
Professional actors	5 (29)
Others	2 (12)

### 3.4 Barriers and challenges

Of the 25 schools that do not use hands‐on US teaching, a lack of US machines, insufficient numbers of trained staff, and time constraints were described as barriers to the use of US in 5 (20%), 9 (36%) and 9 (36%) schools, respectively. Three (12%) schools stated that they were implementing hands‐on US teaching for the first time in the next academic year.

The 23 schools using US to teach anatomy answered a Likert scale question on whether they wished to increase US teaching in their curricula, which yielded a mean score of 4.3/5 (range = 2–5).

### 3.5 Managing incidental findings

Of the 17 schools that use hands‐on US teaching, 15 (88%) have a policy to manage the detection of incidental pathology in subjects. Of the 12 schools that provided summaries of their policies, 11 (92%) outlined that the subject would be advised to consult with their General Practitioner (GP), of which 3 (27%) stated that an accompanying letter would be provided. 2 (17%) only scanned regions with a low incidence of incidental findings, and 3 (25%) schools reported using mandatory consent forms for subjects prior to scanning.

## 4 DISCUSSION

This study is the first to directly explore the extent to which US is integrated within undergraduate anatomy teaching in all UK medical schools. Just over half (55%) of all UK medical schools use US in their anatomy curricula, with the teaching being led by a variety of staff including radiologists, sonographers, and anatomy faculty members. While radiologists are involved in course design in most medical schools that use US in anatomy teaching, they directly deliver teaching in only a minority of these schools. This is significant due to recent recommendations by the Royal College of Radiologists (RCR) (2022), which suggest that radiologists should lead imaging‐based anatomy teaching. Our study has demonstrated that radiologists are involved in the US component of the anatomy curriculum design in 15 out of the 23 medical schools that use US in their anatomy teaching. The recent recommendations by the RCR (2022) may result in a greater collaboration between anatomy faculty members and radiologists, as the new guidance may encourage radiologists who have an interest in teaching to become affiliated with their local anatomy departments.

The integration of US into anatomy teaching at medical schools offers numerous educational benefits. It can facilitate a multimodal learning experience by providing dynamic and real‐time visualization of anatomical structures, which can enhance students' understanding of spatial relationships and anatomical variations. This immediate feedback can promote self‐regulated learning (Zimmerman & Schunk, 2001). Meaningful learning is a prerequisite for transfer of learning (Salomon & Perkins, 1989). In meaningful learning, students should engage in activities that are active, constructive, intentional, authentic, and cooperative (Howland et al., 2011). Using US facilitates meaningful learning as it provides active hands‐on experience and promotes experiential learning, principles advocated by educational theories such as constructivism (Correia et al., 2023; Vygotsky, 1978). Through self‐directed exploration and problem‐solving by interpreting US images, students can engage in metacognitive processes. Although studies examining the impact of US on anatomy learning and cognition are limited, research has shown that the use of US can have a positive impact on memory retention of anatomical information (Dreher et al., 2014; Kondrashov et al., 2015). Implementing US into undergraduate medical education can also cultivate essential motor skills and enhance hand‐eye coordination in students (Nicholls et al., 2014). These acquired proficiencies can serve as fundamental pillars for the acquisition of broader procedural competencies essential for subsequent clinical practice. Furthermore, US is used by a wide range of clinicians such as emergency medicine physicians, anesthetists, cardiologists, and obstetricians. Therefore, earlier exposure to the imaging modality during medical school can benefit doctors throughout their careers.

In this survey, medical schools report that the main challenges to implementing US‐based anatomy teaching are timetabling constraints, the financial costs of US machines, and insufficient training for academic staff. These challenges have been raised by other authors and Patten (2015) also highlights lack of staff training and US equipment as barriers to developing US‐based teaching further (Patten, 2015). Of the schools that do not use hands‐on US teaching, 36% specifically reported time constraints as an issue. There is limited anatomy teaching time in medical school curricula, and therefore schools must prioritize their teaching. One medical school stated that they prioritize “highest yield learning modalities”, which do not include US teaching. In addition, the financial burden of US machines is significant, with costs varying from approximately £6000–19,500 for POCUS devices to £30,000–100,000 for traditional cart‐based scanners (National Institute for Health and Care Excellence, 2021). However, newer hand‐held US transducers are less expensive but may require an additional annual subscription which adds to the financial burden (National Institute for Health and Care Excellence, 2021). With technological advances, such as the development of more affordable and accessible components, the financial burden associated with incorporating US into teaching is expected to decrease in the future. With regard to staffing concerns, many anatomy faculty members do not have a background in radiology. Therefore, additional training sessions are required to ensure they are proficient in providing US‐based teaching.

One potential solution to increase US teaching staff and decrease the student‐to‐teacher ratio, is to implement near‐peer teaching with fewer formally trained supervising sonographers. The student‐to‐teacher ratio is crucial in providing a meaningful learning experience for students, as it allows for more time per student to gain experience in US. With near‐peer teaching, more experienced students are incentivized to teach junior students as it improves their own US abilities while providing them with formal teaching opportunities. Moreover, near‐peer teaching has been shown to be an effective method in improving student performance and confidence through cognitive congruence (Evans & Cuffe, 2009; Viana et al., 2019). At the University of Cambridge, we employ a combination of radiologists, surgeons, clinical anatomists, anatomy demonstrators, and near‐peer teachers to deliver our US‐based anatomy teaching. The non‐radiologists receive US training prior to delivering the teaching. These US sessions are integrated into weekly anatomy teaching sessions and, with the purchase of new portable devices, students are now able to scan in pairs at each session, increasing the time each student can acquire hands‐on US experience. The areas scanned are closely aligned to those at the cadaveric dissection, prosections, and virtual dissection stations to provide a more meaningful learning experience. Based on the concept of a spiral curriculum, a US curriculum is also being developed in our clinical school which builds on the preclinical content.

In addition, a near‐peer model with near‐peer teachers and students as volunteer subjects can help alleviate the financial costs of introducing US‐based anatomy teaching. However, using student volunteers as US subjects creates the potential for incidental findings, which can become a challenge (Dietrich et al., 2022). Potential strategies exist to address this issue, such as pre‐scanning volunteers for pathology in a manner which maintains the individual's dignity and right to confidentiality, or to provide clear guidance upon discovering incidental findings (Smith & Barfoot, 2021). Guidance has been published by the British Medical Ultrasound Society on the use of healthy volunteers for US teaching. Despite this, there is no clear consensus on an optimal policy (British Medical Ultrasound Society, 2018). Most institutions in our survey described directing affected individuals to visit their GP in the event of incidental findings.

Our survey also shows that most UK medical schools wish to increase their use of US in their anatomy departments. The undergraduate radiology syllabus states that students should be aware of imaging features of common pathology, including on US (The Royal College of Radiologists, 2022). The publication of this syllabus may encourage the use of US in anatomy teaching. If students become familiar with normal anatomy on US early in their careers, this could provide a strong foundation for recognizing pathology. The curriculum recommends that anatomy teaching using imaging should be led and governed by radiologists, in collaboration with academic staff in anatomy departments (The Royal College of Radiologists, 2022). This change in the curriculum may act as a driver for medical schools to increase radiologist involvement, which in turn may increase US‐based teaching aiding the implementation of US teaching in the anatomy curriculum. The establishment of a specific forum for US education in the UK would be helpful to share ideas on implementing US‐based anatomy teaching. A similar forum composed of medical schools in California collaborated to develop strategies to help incorporate US education in their respective schools (Chiem et al., 2016). In the UK, an US education forum could liaise with the Medical Schools Council to help drive and coordinate change in US education in UK medical schools.

This study highlights that currently only just over half of all UK medical schools use US in anatomy teaching. We describe the principal challenges involved in integrating US into anatomy teaching and provide suggestions of ways in which these may be overcome. It is hoped that these recommendations can assist institutions in developing the solutions required to comply with the new Undergraduate Radiology Curriculum (2022) (The Royal College of Radiologists, 2022).

## 5 LIMITATIONS OF THE STUDY

This study is based on a survey and the common limitations of surveys include sampling bias, response bias and responses that are limited due to pre‐defined answer choices. Our survey overcame sampling bias due to a 100% response rate giving a fully representative response. The main limitation of our survey was the lack of direct student input as the survey obtained indirect student perceptions. Also, only one staff member at each institution was involved in completing the survey, which may have contributed to response bias. To overcome answers lacking in detail, our survey provided the opportunity for open‐box answers giving survey respondents space to answer questions more freely.

## AUTHOR CONTRIBUTIONS

**Ansh Tandon**: Study design, acquisition of data, data analysis, and drafting of manuscript. **Joseph Moneim**: Acquisition of data, data analysis, and revision of manuscript. **Lauren Hector**: Analysis of data, interpretation of data, and revision of manuscript. **Peter Fletcher**: Data interpretation and critical revision of manuscript. **Ishpal Moonga**: Contributed to study design, data analysis and revision of manuscript. **Sarah Fawcett**: Study design and critical revision of manuscript. **Helen Taylor**: Study design and critical revision of manuscript. **Cecilia Brassett**: Study design and critical revision of manuscript.

## CONFLICT OF INTEREST STATEMENT

The authors declare no conflicts of interest.

## ETHICS STATEMENT

This study has received approval by the Cambridge Human Biology Research Ethics Committee. The ethical approval number is HBREC.2022.10.
